# Serum Procalcitonin to Support Early Triage for Possible Systemic Infection in Patients with Endophthalmitis

**DOI:** 10.3390/diagnostics16091331

**Published:** 2026-04-29

**Authors:** Sun Myung Son, Jae Hyup Lee, Young Jin Kim, Hyun Duck Kwak, Jaewook Yang, Dong Geun Kim

**Affiliations:** Department of Ophthalmology, Inje University Busan Paik Hospital, Busan 47392, Republic of Korea; tjsaudss@naver.com (S.M.S.); dh9746@naver.com (J.H.L.); italia08@naver.com (Y.J.K.); eye.duck23@gmail.com (H.D.K.); oculoplasty@gmail.com (J.Y.)

**Keywords:** endophthalmitis, procalcitonin, systemic infection, inflammatory markers, diagnostic biomarkers, C-reactive protein

## Abstract

**Background:** Endophthalmitis is an ophthalmic emergency in which early identification of concurrent systemic infection is important for appropriate clinical management, yet this distinction is often challenging at presentation. **Methods:** We conducted a retrospective cohort study of patients diagnosed with endophthalmitis at a tertiary referral center between 2017 and 2023. Serum procalcitonin (PCT), C-reactive protein (CRP), white blood cell count, and absolute neutrophil count obtained at presentation were analyzed in relation to clinical classification and systemic infection status, with exploratory receiver operating characteristic (ROC) analyses and Decision Curve Analysis (DCA) used to evaluate diagnostic performance and clinical utility. **Results:** Among 152 patients, serum inflammatory marker levels were significantly higher in patients classified as having endogenous endophthalmitis than in exogenous cases (*p* < 0.01), with the greatest separation observed for PCT and CRP. In ROC analyses, PCT demonstrated greater discriminatory capacity for concurrent systemic infection than other markers, with a sensitivity of 91.8%, specificity of 97.9%, and an area under the curve (AUC) of 0.964 at an ROC-derived threshold of 0.11 ng/mL. CRP also showed high discriminatory performance (AUC 0.947), whereas white blood cell count and absolute neutrophil count showed lower AUC values. In patients presenting with endophthalmitis and concurrent uncontrolled systemic infection, PCT showed a higher AUC than CRP (0.995 vs. 0.939). Furthermore, DCA demonstrated that a comprehensive model combining inflammatory biomarkers with clinical risk factors provided the highest net benefit for clinical triage. **Conclusions:** These findings suggest that serum PCT, particularly when integrated into a multidimensional clinical assessment, may serve as a valuable adjunctive tool to support early triage when systemic infection is a concern.

## 1. Introduction

Endophthalmitis is a bacterial or fungal infection of the eye involving the vitreous or aqueous humor and is typically an acute ophthalmic emergency that can lead to irreversible vision loss. It is most commonly caused by exogenous sources such as intraocular surgery, penetrating ocular trauma, or infectious keratitis [1]. In contrast, endogenous endophthalmitis results from hematogenous spread of pathogens from systemic infections and accounts for approximately 2–8% of all cases [2,3,4,5,6].

As exogenous causes such as surgery or trauma are usually well-documented and infrequent, clinical history often facilitates the differential diagnosis of endophthalmitis. Nevertheless, diagnostic challenges may arise when patients present with ambiguous ocular history or coexisting systemic conditions. With the increasing use of intravitreal injections (IVIs) for retinal diseases. [7,8] distinguishing between exogenous and endogenous endophthalmitis has become more complex, particularly in elderly patients receiving long-term treatment. Therefore, when such patients present with endophthalmitis, it is essential to consider underlying systemic conditions that may predispose them to endogenous infection.

Serum procalcitonin (PCT), a precursor of calcitonin, is a well-established biomarker of systemic bacterial infection. It is typically absent in healthy individuals and less affected by local inflammation or non-infectious stimuli compared to C-reactive protein (CRP) [9,10]. These characteristics suggest that serum procalcitonin may provide supportive information regarding the likelihood of concurrent systemic infection in patients presenting with endophthalmitis. However, despite extensive evidence from internal medicine, the role of procalcitonin in ophthalmic infections remains unclear, and its potential advantage over conventional inflammatory markers such as CRP has not been systematically evaluated in endophthalmitis. Therefore, this study aimed to evaluate the clinical relevance of serum procalcitonin in patients with endophthalmitis, with a particular focus on its association with concurrent systemic infection and its potential role in supporting early clinical triage.

## 2. Materials and Methods

This retrospective cohort study included a consecutive series of patients diagnosed with endophthalmitis at Inje University Busan Paik Hospital, a tertiary referral center, between March 2017 and June 2023. The diagnosis of endophthalmitis was established based on clinical findings by treating ophthalmologists. Serologic tests, including serum procalcitonin (PCT), C-reactive protein (CRP), erythrocyte sedimentation rate (ESR), white blood cell count (WBC), and absolute neutrophil count (ANC), were performed at initial presentation at the discretion of the treating physician. To allow for comparative analysis, only patients with at least two available serologic markers were included. The study was conducted in accordance with the Declaration of Helsinki, and approved by the Institutional Review Board of Inje University Busan Paik Hospital (BPIRB 2023-08-025). The requirement for informed consent was waived due to the retrospective nature of the study.

WBC and ANC were measured using the Sysmex XN-9100™ system (Sysmex, Kobe, Japan) with fluorescence-based reagents, including Lysercell WNR, Fluorocell WNR, Stromatolyser-4DL, and Fluorocell WDF. ESR was determined using the Test 1 analyzer (Alifax Srl, Polverara, Italy). CRP levels were analyzed with the Hitachi LABOSPECT 008 (Hitachi High-Tech Co., Tokyo, Japan) using QUALIGENT CRP reagents (Sekisui Medical Co., Ltd., Tokyo, Japan). PCT was measured using the VIDAS system with Vidas Brahms PCT^®^ reagents (both from bioMérieux, Marcy-l’Etoile, France).

Demographic data, including age, sex, laterality, source of infection, interval between the suspected infection onset and ophthalmic presentation, causative microorganisms, and treatment details were systematically collected. Ophthalmic data, including best-corrected visual acuity (BCVA) and intraocular pressure (IOP), were also recorded. In bilateral cases, the eye with worse BCVA was used for analysis. The diagnostic value of each serological test in differentiating endogenous from exogenous endophthalmitis was evaluated. For patients with very low vision, visual acuity was converted to logarithm of the minimum angle of resolution (logMAR) using standard approximations: counting fingers = 1.9, hand motion = 2.3, light perception = 2.7, and no light perception = 3.0 [11]. As this was an exploratory retrospective study, a formal a priori power calculation was not performed. Instead, the sample size was determined by the total number of consecutive eligible patients who presented during the defined study period to maximize the available data for analysis.

Patients with endogenous endophthalmitis were categorized into two subgroups according to the activity of their primary systemic source at the time of presentation. A controlled systemic infection was operationally defined as a case where specialized inpatient treatment for the primary systemic infection (e.g., liver abscess, pneumonia) had been officially completed, and the patient was no longer receiving systemic antimicrobial therapy upon referral to the ophthalmology clinic. In contrast, an uncontrolled systemic infection was defined as any presentation occurring during the active, symptomatic phase of the systemic disease requiring ongoing systemic treatment. Given the heterogeneous referral pathways from various primary and secondary hospitals, the clinical status of ‘control’ was determined using the patient’s actual treatment trajectory (successful hospital discharge and cessation of systemic antibiotics) as a clinically integrated surrogate for biological and infectious stability.

The clinical utility and added value of serum biomarkers were evaluated using Decision Curve Analysis (DCA). Net benefit was calculated across a range of threshold probabilities by subtracting the proportion of false-positive results (weighted by the relative harm of an unnecessary systemic workup) from the proportion of true-positive results. The underlying clinical assumption of this model is that the harm of missing a true concurrent systemic infection (false negative) outweighs the relatively lower risks and costs associated with an unnecessary systemic diagnostic workup (false positive), which guides the clinical interpretability of the threshold probabilities. We compared the following diagnostic strategies: (1) standalone biomarker models (PCT or CRP alone), (2) a clinical phenotype-only model (incorporating age, initial visual acuity, diabetes mellitus, immunocompromised status, and ocular severity indices), (3) combined biomarker-clinical models, and (4) a comprehensive ‘Full Model’ integrating all biomarkers and clinical variables. The ‘Treat All’ (evaluating every patient for systemic infection) and ‘Treat None’ strategies served as reference benchmarks.

Continuous variables were tested for normality using the Shapiro–Wilk test. Variables following a normal distribution were expressed as means ± standard deviations and compared using independent *t*-tests. Non-normally distributed variables were presented as medians with interquartile ranges [Q1–Q3] and compared using the Wilcoxon rank-sum test. Categorical variables were presented as frequencies and percentages and compared using Pearson’s chi-square test or Fisher’s exact test, as appropriate. In addition, the Kolmogorov–Smirnov test was used to assess the distributional differences in each serologic marker between the endogenous and exogenous groups. Receiver operating characteristic (ROC) curves were constructed for each serological marker to assess diagnostic performance. Optimal cut-off values were determined using the Youden index, defined as the point maximizing the sum of sensitivity and specificity. The corresponding area under the curve (AUC) was also calculated for each test. To ensure the internal validity of the diagnostic models and to assess the risk of overfitting, we performed internal validation using a non-parametric bootstrapping procedure. For each inflammatory marker, both the apparent area under the receiver operating characteristic curve (AUC) and the optimism-corrected AUC were calculated. The degree of optimism was estimated by calculating the average difference between the AUC of the bootstrap models and the AUC of those models applied to the original dataset. All statistical analyses were conducted using R software version 4.1.3 (R Foundation for Statistical Computing, Vienna, Austria), with statistical significance set at *p* < 0.05.

## 3. Results

### 3.1. Baseline Patient Characteristics

A total of 186 patients were diagnosed with endophthalmitis during the study period. Of these, 34 patients (2 with endogenous and 32 with exogenous endophthalmitis) were excluded due to a lack of serologic testing. Consequently, 152 patients were included in the analysis. This cohort consisted of 66 patients with endogenous endophthalmitis and 86 with exogenous endophthalmitis. The clinical characteristics and etiologies of the excluded and included patients are compared in Supplementary Table S1.

Baseline demographic and clinical characteristics are summarized in Table 1. There were no significant differences between the two groups with respect to age, sex distribution, best-corrected visual acuity, intraocular pressure, or primary treatment modality. Bilateral involvement was significantly more frequent in the endogenous group than in the exogenous group (42.4% vs. 2.3%, *p* < 0.001). The proportion of patients in whom the onset of the causative infection could not be identified was also higher in the endogenous group (45.5% vs. 5.8%, *p* < 0.001). Among patients with an identifiable causative event, the interval from the suspected infection to the diagnosis of endophthalmitis was shorter in endogenous cases than in exogenous cases (7.4 ± 6.0 days vs. 11.4 ± 12.9 days, *p* = 0.023). Detailed information on causative infections and pathogens is provided in Supplementary Table S2.

Due to the retrospective nature of the study, inflammatory biomarkers were not uniformly assessed in all patients at the time of presentation; the specific counts and proportions of available measurements for each biomarker are summarized in Table 2. Similar to the trends observed among excluded patients, indicators such as serum procalcitonin (PCT) and C-reactive protein (CRP) were measured at a significantly lower frequency in the exogenous cohort compared to the endogenous cohort (PCT: 55.8% vs. 74.2%, *p* = 0.030; CRP: 62.8% vs. 97.0%, *p* < 0.001).

### 3.2. Distribution of Serum Inflammatory Markers

Serum levels of procalcitonin (PCT), C-reactive protein (CRP), white blood cell (WBC) count, and absolute neutrophil count (ANC) at presentation were compared between patients classified as having endogenous and exogenous endophthalmitis. All four inflammatory markers showed significantly higher values in the endogenous group than in the exogenous group (PCT, *p* < 0.001; CRP, *p* < 0.001; WBC, *p* = 0.002; ANC, *p* < 0.001).

**Figure 1:**
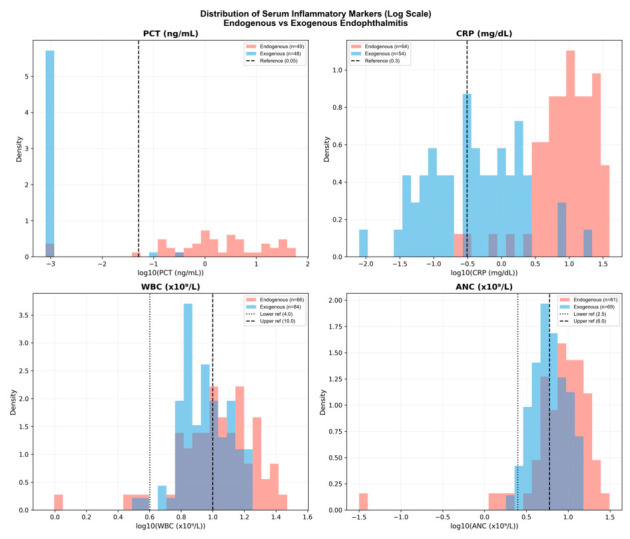
Distributions of serum inflammatory marker levels in endogenous and exogenous endophthalmitis. The concentrations of procalcitonin (PCT), C-reactive protein (CRP), white blood cell (WBC) count, and absolute neutrophil count (ANC) are presented on a logarithmic scale (y-axis) to better visualize the heavily skewed data and improve visibility of differences at lower concentration ranges. Horizontal dashed lines indicate the diagnostic thresholds for each marker. All four markers were significantly higher in the endogenous group compared to the exogenous group (PCT, *p* < 0.001; CRP, *p* < 0.001; WBC, *p* = 0.007; ANC, *p* < 0.001). PCT and CRP exhibited markedly greater intergroup separation (PCT D = 0.898; CRP D = 0.866) than WBC and ANC (ANC D = 0.345; WBC D = 0.269) based on the Kolmogorov–Smirnov test. While mild elevations in PCT (0.05–0.5 ng/mL) were observed in sporadic exogenous cases, no markedly elevated PCT values were noted in the exogenous group.

Distributional differences between the two groups were further evaluated using the Kolmogorov–Smirnov test. Among the four markers, PCT showed the greatest separation between endogenous and exogenous cases (D = 0.898, *p* < 0.001), followed by CRP (D = 0.866, *p* < 0.001). In contrast, ANC and WBC demonstrated more modest separation (ANC: D = 0.345, *p* = 0.001; WBC: D = 0.269, *p* = 0.007). The distributions of serum inflammatory markers in the two groups are illustrated in Figure 1.

### 3.3. Comparative Performance of Inflammatory Markers

The discriminatory performance of serum inflammatory markers for concurrent systemic infection was evaluated using receiver operating characteristic (ROC) analyses. Among the four markers assessed, serum procalcitonin (PCT) demonstrated the highest overall discriminatory capacity. At an ROC-derived threshold of 0.11 ng/mL, PCT showed a sensitivity of 91.8% and specificity of 97.9%, with an area under the curve (AUC) of 0.964.

**Figure 2:**
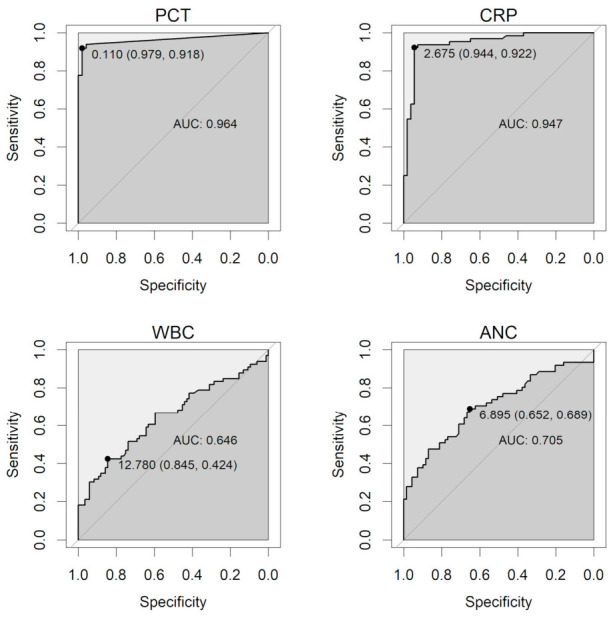
ROC curves of four serologic markers for differentiating endogenous from exogenous endophthalmitis. Markers include procalcitonin (PCT, ng/mL), C-reactive protein (CRP, mg/dL), white blood cell (WBC) count, and absolute neutrophil count (ANC) (both in ×10^3^/μL). Each curve indicates the optimal cut-off value with specificity and sensitivity presented as cut-off (specificity, sensitivity). PCT and CRP demonstrated higher diagnostic accuracy (AUC = 0.964 and 0.947, respectively) compared with ANC (0.705) and WBC (0.646).

C-reactive protein (CRP) also showed high discriminatory performance, with an AUC of 0.947 at an optimal cut-off value of 2.87 mg/dL, although its specificity was lower when conventional reference ranges were applied. In contrast, white blood cell (WBC) count and absolute neutrophil count (ANC) demonstrated more limited discriminatory capacity, with AUC values of 0.646 and 0.705, respectively. Detailed diagnostic performance metrics for each marker, including sensitivity, specificity, and predictive values at both reference-based and ROC-derived thresholds, are summarized in Table 3, and corresponding ROC curves are shown in Figure 2.

### 3.4. Subgroup Analysis in Patients with Uncontrolled Systemic Infection

A subgroup analysis was performed to evaluate the performance of serum inflammatory markers in patients presenting with endophthalmitis and concurrent uncontrolled systemic infection at the time of ophthalmic evaluation. In this subgroup, serum procalcitonin (PCT) demonstrated a higher discriminatory capacity than other inflammatory markers. The area under the curve (AUC) for PCT was 0.995, indicating near-complete separation, whereas C-reactive protein (CRP) showed a lower AUC of 0.939. The corresponding diagnostic performance metrics for serum inflammatory markers in this subgroup are summarized in Table 4.

Internal validation through bootstrapping (1000 resamples) yielded optimism-corrected AUC values that were nearly identical to the apparent AUCs, particularly for PCT (corrected AUC 0.9639 in Table 3 and 0.9946 in Table 4). This minimal optimism confirms the robustness and high reproducibility of serum PCT as a diagnostic tool in our study population.

**Figure 3:**
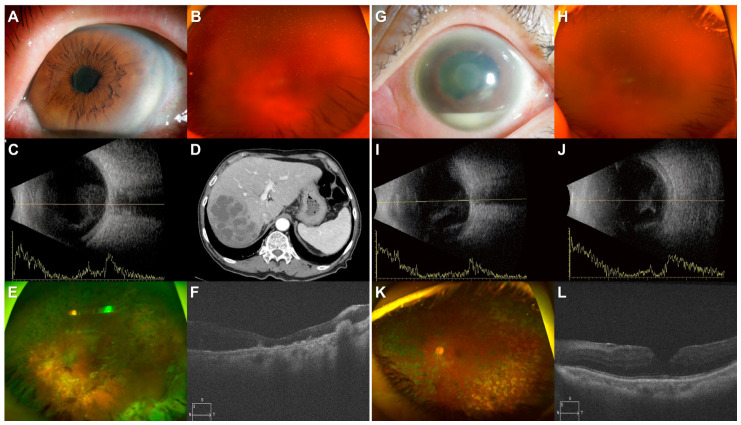
Representative Cases of Endogenous and Exogenous Endophthalmitis.

Representative cases comparing exogenous endophthalmitis and occult liver abscess-associated endogenous endophthalmitis are presented in Figure 3.

Case 1 (Figure 3A–F): An 87-year-old male with diabetes presented with panuveitis 6 days after an intravitreal bevacizumab injection. (Figure 3A) Slit-lamp examination reveals a dense pupillary membrane and 4+ anterior chamber cells. (Figure 3B) Fundus photography shows severe vitreous opacity. (Figure 3C) B-scan ultrasonography demonstrates high-amplitude intravitreal echoes. (Figure 3D) Systemic evaluation revealed elevated inflammatory markers (Procalcitonin: 1.3 ng/mL, CRP: 13.02 mg/dL), and abdominal CT confirmed a hepatic abscess. After treatment for Klebsiella pneumoniae sepsis, pars plana vitrectomy (PPV) was performed on day 10. (Figure 3E) Postoperative fundus photography shows attached and atrophic retina following focal infiltration. (Figure 3F) Postoperative OCT image shows macular atrophy and subretinal old infiltrations. Final BCVA was hand motion.

Case 2 (Figure 3G–L): A 57-year-old female with PDR presented 2 days after a intravitreal bevacizumab injection. (Figure 3G) Slit-lamp and fundus examinations show a significant hypopyon, fibrous membrane. (Figure 3H) Fundus photography shows dense vitritis. (Figure 3I,J) B-scan ultrasonography reveals diffuse high-reflectivity echoes in the vitreous cavity. In contrast to Case 1, systemic inflammatory markers were normal procalcitonin (<0.05 ng/mL) and mildy elevated CRP (0.53 mg/dL), with no systemic infection focus. Following emergent PPV, Bacillus cereus was identified. (Figure 3K) At 1-month follow-up, fundus photography shows relatively stable retina and (Figure 3L) OCT image shows a well-restored macular structure. Final BCVA was 0.6 in Snellen visual acuity.

### 3.5. Clinical Utility Evaluation via Decision Curve Analysis

**Figure 4:**
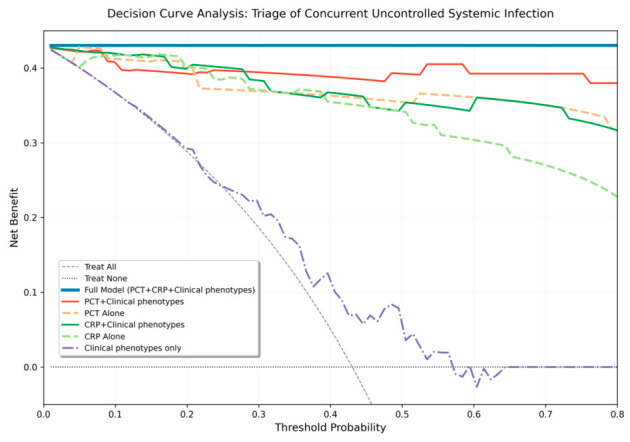
Decision Curve Analysis (DCA) for the triage of concurrent uncontrolled systemic infection in patients with endophthalmitis. The y-axis represents net clinical benefit, and the x-axis represents the range of threshold probabilities used for deciding on an urgent systemic evaluation. The horizontal dotted line represents a ‘Treat None’ (target no one) strategy, and the dashed gray line represents a ‘Treat All’ (evaluate everyone) strategy. Standalone procalcitonin (PCT) and C-reactive protein (CRP) models (dashed lines) provide a consistent net benefit over the clinical phenotype-only model (dot-dashed line). The Full Model (solid blue line), which combines both biomarkers with seven baseline clinical and systemic risk factors, yields the highest net benefit across all clinically appropriate threshold probabilities, illustrating its superior value as a comprehensive triage tool.

Decision Curve Analysis was performed to determine the clinical utility of serum biomarkers in the triage of concurrent uncontrolled systemic infection (Figure 4). Both PCT-based and CRP-based strategies demonstrated a consistent and substantial net benefit over the default ‘Treat All’ and ‘Treat None’ strategies across a wide and clinically relevant range of threshold probabilities (approximately 0.10 to 0.80). While single-marker models provided excellent discriminatory utility, the composite Full Model—integrating PCT, CRP, and seven baseline clinical factors (age, diabetes, immunocompromised status, initial BCVA, and slit-lamp examination findings like corneal ulcer, hypopyon and panophthalmitis)—achieved the highest overall net benefit across the entire threshold spectrum. This suggests that a multidimensional triage approach, amalgamating rapid serological biomarkers with clinical risk factors, most effectively identifies critical systemic cases while minimizing unnecessary and invasive systemic evaluations in localized exogenous infections.

## 4. Discussion

Identifying the presence of concurrent systemic infection in patients with endophthalmitis is clinically important but can be challenging when ocular or systemic clues are limited. The reported rate of delayed or initially incorrect diagnosis of endogenous endophthalmitis ranges from 16% to 63% [4]. This diagnostic uncertainty complicates early clinical decision-making, particularly when determining the need for systemic evaluation alongside prompt intraocular treatment. Therefore, this study aimed to investigate the clinical relevance of serum biomarkers that could complement conventional assessment and support early clinical triage in patients presenting with endophthalmitis.

In our study, both CRP and PCT levels were significantly higher in patients classified as having endogenous endophthalmitis compared to exogenous cases; however, their clinical implications appeared to differ. While our optimized PCT threshold (0.11 ng/mL) aligns with established clinical ranges (0.05–0.5 ng/mL) in diverse infectious diseases, [12,13,14] the derived CRP threshold (2.87 mg/dL) deviates significantly from its standard reference limit (0.3 mg/dL), which markedly compromised specificity when applied to our cohort. PCT showed greater discriminatory capacity in relation to active systemic infection and was more closely associated with the presence of ongoing, uncontrolled infection. Furthermore, the diagnostic value remained consistent when we validated our data against PCT criteria from previous literature, showing similar sensitivity and specificity (Supplementary Table S3). Schuetz et al. demonstrated that PCT-guided management improved antibiotic stewardship in lower respiratory tract infections, reflecting its close association with infection control status [15]. Similarly, a large-scale meta-analysis by Wacker et al. reported that PCT outperformed CRP in both diagnostic specificity and infection monitoring [16]. Unlike CRP, which may remain elevated after bacterial eradication, PCT levels typically decline rapidly with effective treatment, supporting its role as a marker of active infection rather than residual inflammation [17].

False-positive elevations of serum inflammatory markers were most frequently observed in patients with exogenous endophthalmitis associated with corneal ulcers. Among the 12 patients in whom CRP was measured, 7 (58.3%) showed elevated CRP levels despite the absence of systemic infection, including two patients who progressed to panophthalmitis and required primary evisceration. False-positive elevations of PCT were less common, observed in 2 of 11 patients (18.2%). These findings may be related to disruption of the blood–ocular barrier in severe corneal infections, which can lead to systemic inflammatory responses without true hematogenous dissemination. Although PCT is generally considered less responsive to localized infections than CRP, previous studies have reported mild PCT elevations in conditions such as septic arthritis, osteomyelitis, pyelonephritis, and pancreatitis [18,19,20,21]. Accordingly, this intermediate range is often interpreted with caution and considered suggestive of localized, rather than systemic, infection [22,23]. Taken together, these findings suggest that similar interpretative considerations may apply in ophthalmic infections, particularly in cases of exogenous endophthalmitis associated with corneal ulceration. Accordingly, mildly elevated PCT levels should be interpreted with caution and not be considered definitive evidence of uncontrolled systemic infection in this setting. Additional studies in larger cohorts would help further clarify the reliability of serum biomarkers when the blood–ocular barrier is compromised.

**Figure 5:**
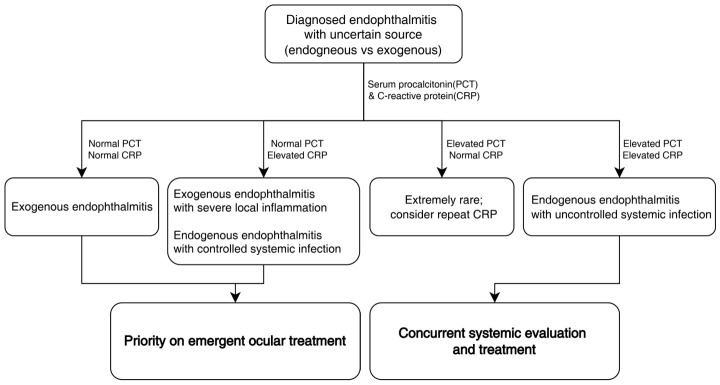
Proposed interpretative framework for serum PCT and CRP in patients with endophthalmitis. Proposed interpretative framework illustrating how serum procalcitonin (PCT) and C-reactive protein (CRP) levels may be considered in patients presenting with endophthalmitis when the source of infection is uncertain. The framework summarizes patterns observed in the present study and is intended to support clinical interpretation rather than to serve as a diagnostic or management algorithm. Serum biomarkers should be interpreted in conjunction with clinical findings and comprehensive ophthalmic and systemic evaluation.

PCT levels are known to respond dynamically to changes in systemic infection activity and to decrease rapidly with effective treatment [24,25]. This makes PCT a useful tool for assessing treatment effectiveness and determining whether antibiotics can be safely discontinued in systemic infections [22,23,26]. In this study, three patients with endogenous endophthalmitis showed false-negative PCT results; however, all had systemic infections that were already controlled with antibiotic therapy at the time of ophthalmic presentation, while CRP levels remained elevated. These observations suggest that normal PCT levels do not necessarily exclude a history of systemic infection but may indicate adequate infection control. In the clinical setting, where blood tests are primarily performed to assess the presence of uncontrolled systemic infection, serum PCT may provide complementary information when interpreted alongside clinical findings and other inflammatory markers. Based on these observations, we present a conceptual framework that may assist in the interpretation of serum PCT and CRP levels in selected clinical scenarios where the source of infection is uncertain (Figure 5).

Overall, the diagnostic performance of serum PCT and CRP observed in the present study was higher than that reported in internal medicine literature. In a widely cited meta-analysis comparing the diagnostic value of PCT and CRP for bacterial infection, Simon et al. demonstrated the superiority of PCT over CRP, reporting an overall sensitivity of 88% versus 75% and specificity of 81% versus 67%, respectively [27]. Subsequently, a meta-analysis by Wacker et al. reported a pooled sensitivity of 77%, specificity of 79%, and an area under the curve of 0.85 for PCT in the diagnosis of sepsis across heterogeneous clinical populations [16]. We postulate that this difference may be related to the nature of the clinical comparison. Unlike systemic sepsis cohorts that compare broad infectious sources against diverse non-infectious inflammatory “noise,” our ophthalmic triage setting evaluates a more constrained population. We fundamentally compare patients with “systemic infection plus metastatic eye infection” against those with “eye infection in a healthy systemic state.” Furthermore, rather than representing a continuum of infection severity with frequent transitional states, endogenous and exogenous endophthalmitis are etiologically distinct entities. This reduced etiological noise and lack of intermediate states likely strengthen the biomarker signal and reduce diagnostic overlap. Nevertheless, such near-complete separation and unusually high AUC values are uncommon in heterogeneous real-world settings. Therefore, these exceptional performance metrics should be interpreted with caution and framed as exploratory results that strictly reflect the specific characteristics of this selected cohort. These findings should be further examined and validated in larger and prospective studies.

Translating this high diagnostic accuracy into practical decision-making, our decision curve analysis further highlights the clinical utility of a comprehensive triage model. While continuous standalone PCT thresholds effectively discriminated against concurrent uncontrolled systemic infection, the composite ‘Full Model’—which integrates inflammatory biomarkers with baseline clinical risk factors such as age, immunocompromised status, and typical slit-lamp findings—consistently provided the highest net benefit across all clinically relevant threshold probabilities. This multidimensional approach addresses the inherent complexity of ophthalmic triage, suggesting that rather than relying solely on a single laboratory value, integrating serological data with patient-specific risk profiles can systematically optimize care. Practically, this strategy maximizes the detection of high-risk endogenous cases while concurrently minimizing the clinical and financial burden of unnecessary systemic workups for localized, exogenous infections.

Beyond the etiological dichotomy, the retrospective nature of our study resulted in a significant proportion of patients being excluded and a relatively low rate of biomarker measurement among the included participants, leading to a limited sample size for the diagnostic performance analysis. As shown in Table 2, the higher testing rates in the endogenous group likely reflect the treating physicians’ clinical suspicion of underlying systemic disease. This inherent selection bias must be explicitly acknowledged, as it may influence and potentially inflate the reported diagnostic performance. However, as demonstrated in Supplementary Tables S1 and S2, the majority of cases where biomarkers were not assessed represented clinically certain and relatively mild (better initial and final BCVA) exogenous infections. Given that these omitted tests were primarily in patients with a very low pre-test probability of systemic involvement, we anticipate that the potential for overestimating the diagnostic value due to the exclusion of these milder presentations is likely minimal; nevertheless, future validation in larger, consecutively enrolled longitudinal cohorts is necessary to definitively confirm these high performance metrics.

This study has several limitations. First, its retrospective design and reliance on medical records introduce the potential for selection bias, particularly with respect to the decision to obtain serologic tests at presentation. Second, serologic markers were not measured uniformly across all patients, which limited direct comparison of all inflammatory markers within the entire cohort. Third, the relatively small sample size, including a limited number of patients with specific clinical scenarios (such as false-positive or false-negative results), restricted the use of robust statistical methods, such as multivariate and more detailed subgroup analyses. Fourth, our operational definition of ‘uncontrolled systemic infection’ was based on ongoing treatment status rather than direct measures of biological activity. Given the rapid kinetics of procalcitonin, this reliance on treatment history may introduce misclassification related to the exact timing of biomarker measurement. Furthermore, the inability to obtain precise medical histories regarding the start and end times of prior systemic antibiotic therapy precluded further detailed analysis of its effects on biomarker profiles. In addition, as this was a single-center study, the generalizability of the findings to other clinical settings may be limited. Therefore, the observed diagnostic performance of serum biomarkers should be interpreted cautiously. Nevertheless, despite these limitations, this study provides an initial and clinically relevant perspective on the interpretation of serum procalcitonin in ophthalmic infections, an area that has been minimally explored to date.

Building upon these initial findings, future research should focus on several key areas. Primarily, there is a clear need for prospective, multi-center validation studies utilizing the interpretive framework proposed in this study to firmly establish its clinical utility. Furthermore, future investigations must prioritize elucidating the precise kinetic relationship between prior systemic antibiotic administration and subsequent serum procalcitonin levels. Finally, there is significant potential to expand this triage model to other acute ocular infections with suspicious systemic associations, aiming to further refine our ability to identify occult infectious sources in the emergency setting.

## 5. Conclusions

Serum PCT, particularly when integrated into a multidimensional clinical assessment alongside patient risk factors, may serve as a valuable adjunctive tool for early triage in patients presenting with endophthalmitis. When definitive ocular or systemic clues are limited, this comprehensive approach systematically reduces the risk of missing critical, uncontrolled systemic infections while concurrently minimizing the cost, time, and patient burden associated with unnecessary systemic evaluations in localized exogenous cases. To further validate and refine this diagnostic model, future studies elucidating the underlying mechanisms of rare false-positive and false-negative presentations remain necessary.

## Figures and Tables

**Table 1:** Comparison of the clinical characteristics between endogenous and exogenous Endophthalmitis.

	Endogenous Endophthalmitis (*n* = 66)	Exogenous Endophthalmitis (*n* = 86)	*p*-Value
Age	66.9 ± 11.7	66.1 ± 13.6	0.710
Male, *n* (%)	33 (50.0%)	49 (57.0%)	0.489
Diabetes Mellitus	25 (37.9%)	29 (33.7%)	0.612
Immunocompromised	6 (9.1%)	5 (5.8%)	0.533
Bilateral involvement	28 (42.4%)	2 (2.3%)	<0.001
Best-corrected visual acuity (logMAR) ^1^	1.7 ± 1.0	1.8 ± 1.0	0.576
Intraocular pressure, mmHg ^1^	20.6 ± 11.0	24.1 ± 11.7	0.094
Hypopyon	30 (45.5%)	49 (57.0%)	0.213
Panophthalmitis	4 (6.1%)	10 (11.6%)	0.372
Corneal ulcer	3 (4.5%)	14 (16.3%)	0.044
Duration after the causative infection			
Unknown	30 (45.5%)	5 (5.8%)	<0.001
Known	36 (54.5%)	81 (94.2%)	
Mean (days)	7.4 ± 6.0	11.4 ± 12.9	0.023
<7 days	18 (50.0%)	44 (53.0%)	0.074
7–30 days	17 (47.2%)	25 (30.1%)	
≥30 days	1 (2.8%)	12 (14.8%)	
Main treatment			0.081
Vitrectomy	35 (53.0%)	51 (66.3%)	
Primary evisceration	2 (3.0%)	8 (9.3%)	
Intravitreal antibiotics	12 (18.2%)	11 (12.8%)	
Systemic and topical antibiotics	11 (16.7%)	7 (8.1%)	
Treatment refusal or follow-up loss	6 (9.1%)	3 (3.5%)	

^1^ Bilateral cases were evaluated based on measurements in the worse eye.

**Table 2:** Availability and measurement frequency of serum inflammatory biomarkers.

Inflammatory Biomarker	Assessed Patients, *n* (%)	Endogenous Group, *n* (%)	Exogenous Group, *n* (%)	*p*-Value
PCT	97 (63.8%)	49 (74.2%)	48 (55.8%)	0.030
CRP	118 (77.6%)	64 (97.0%)	54 (62.8%)	<0.001
WBC	150 (98.7%)	66 (100.0%)	84 (97.7%)	0.505
ANC	130 (85.5%)	61 (92.4%)	69 (80.2%)	0.059
ESR	36 (23.7%)	12 (18.2%)	24 (27.9%)	0.228

**Table 3:** Diagnostic performance of serologic tests in differentiating endogenous from exogenous endophthalmitis.

Test	Cut-Off Criterion	Sensitivity, (*n*/*N*) [95% CI]	Specificity, (*n*/*N*) [95% CI]	PPV, (*n*/*N*) [95% CI]	NPV, (*n*/*N*) [95% CI]	AUC [95% CI]	Optimism-Corrected AUC [95% CI]
PCT	Normal range (<0.05 ng/mL)	93.9% (46/49) [83.5–97.9)	95.8% (46/48) [86.0–98.8]	95.8% (46/48) [86.0–98.8]	93.9% (46/49) [83.5–97.9]	-	
	ROC cut-off (0.11 ng/mL)	91.8% (45/49) [80.8–96.8]	97.9% (47/48) [89.1–99.6]	97.8% (45/46) [88.7–99.6]	92.2% (47/51) [81.5–96.9]	0.9643 [0.9252–0.9953]	0.9643 [0.9252–1.0]
CRP	Normal range (≤0.3 mg/dL)	98.4% (63/64) [91.7–99.7]	40.7% (22/54) [28.7–54.0]	66.3% (63/95) [56.3–75.0]	95.7% (22/23) [79.0–99.2]	-	
	ROC cut-off (2.87 mg/dL)	92.2% (59/64) [83.0–96.6]	94.4% (51/54) [84.9–98.1]	95.2% (59/62) [86.7–98.3]	91.1% (51/56) [80.7–96.1]	0.9466 [0.8954–0.9825]	0.9467 [0.9108–0.9978]
WBC	Normal range (4.0~10.0 × 10^9^/L)	59.1% (39/66) [47.0–70.1]	64.3% (54/84) [53.6–73.7]	56.5% (39/69) [44.8–67.6]	66.7% (54/81) [55.9–76.0]	-	
	ROC cut-off (12.86 × 10^9^/L)	42.4% (28/66) [31.2–54.4]	84.5% (71/84) [75.3–90.7]	68.3% (28/41) [53.0–80.4]	65.1% (71/109) [55.8–73.4]	0.6462 [0.5547–0.7339]	0.6463 [0.5584–0.7377]
ANC	Normal range (2.5~6.0 × 10^9^/L)	73.8% (45/61) [61.6–83.2]	50.7% (35/69) [39.2–62.2]	57.0% (45/79) [46.0–67.3]	68.6% (35/51) [55.0–79.7]	-	
	ROC cut-off (9.93 × 10^9^/L)	47.5% (29/61) [35.5–59.8]	87.0% (60/69) [77.0–93.0]	76.3% (29/38) [60.8–87.0]	65.2% (60/92) [55.1–74.2]	0.7046 [0.6092–0.7898]	0.7039 [0.6194–0.7999]

ANC, absolute neutrophil count; AUC, area under the curve; CRP, C-reactive protein; NPV, negative predictive value; PCT, procalcitonin; PPV, positive predictive value; WBC, white blood cell count.

**Table 4:** Diagnostic performance of serologic tests in identifying endogenous endophthalmitis with uncontrolled systemic Infection.

Test	Cut-Off Criterion	Sensitivity, (*n*/*N*) [95% CI]	Specificity, (*n*/*N*) [95% CI]	PPV, (*n*/*N*) [95% CI]	NPV, (*n*/*N*) [95% CI]	AUC [95% CI]	Optimism-Corrected AUC [95% CI]
PCT	Normal range (<0.05 ng/mL)	100.0 (44/44) [92.0–100.0]	92.5 (49/53) [82.1–97.0]	91.7 (44/48) [80.4–96.7]	100.0 (49/49) [92.7–100.0]		
	ROC cut–off (0.05 ng/mL)	100.0 (44/44) [92.0–100.0]	92.5 (49/53) [82.1–97.0]	91.7 (44/48) [80.4–96.7]	100.0 (49/49) [92.7–100.0]	0.9946 [0.9838–1.0]	0.9946 [0.9838–1.0]
CRP	Normal range (≤0.3 mg/dL)	98.3 (58/59) [91.0–99.7]	37.3 (22/59) [26.1–50.0]	61.1 (58/95) [51.0–70.2]	95.7 (22/23) [79.0–99.2]		
	ROC cut–off (2.87 mg/dL)	93.2 (55/59) [83.8–97.3]	88.1 (52/59) [77.5–94.1]	88.7 (55/62) [78.5–94.4]	92.9 (52/56) [83.0–97.2]	0.9387 [0.8865–0.9763]	0.9385 [0.8865–0.9763]
WBC	Normal range (10.0 × 10^9^/L)	60.7% (37/61) [48.1–71.9]	61.8% (57/89) [53.7–73.2]	53.6 (40/74) [42.8–64.9]	70.4 (55/76) [61.4–81.2]		
	ROC cut–off (12.86 × 10^9^/L)	45.9% (28/61) [34.0–58.3]	85.4% (76/89) [76.6–91.3]	68.3% (28/41) [53.0–80.4]	69.7% (76/109) [60.5–77.6]	0.6581 [0.5616–0.7521]	0.6569 [0.5616–0.7521]
ANC	Normal range (6.0 × 10^9^/L)	76.8% (43/56) [64.2–85.9]	51.4% (38/74) [40.2–62.4]	54.4% (43/79) [43.5–65.0]	74.5% (38/51) [61.1–84.5]		
	ROC cut–off (9.93 × 10^9^/L)	50.0% (28/56) [37.3–62.7]	86.5% (64/74) [76.9–92.5]	73.7% (28/38) [58.0–85.0]	69.6% (64/92) [59.5–78.0]	0.7253 (0.6262–0.8114]	0.7251 (0.6392–0.8243]

ANC, absolute neutrophil count; AUC, area under the curve; CRP, C-reactive protein; NPV, vegative predictive value; PCT, procalcitonin; PPV, positive predictive value; WBC, white blood cell count.

## Data Availability

Data are contained within the article. The additional data presented in this study are available on request from the corresponding author.

## Supplementary Materials

The following supporting information can be downloaded at: https://www.mdpi.com/article/10.3390/diagnostics16091331/s1, Table S1: Comparison of Included vs. Excluded Patients, Table S2: Detailed information on the causative infections and pathogens, Table S3: Diagnostic Performance of Procalcitonin at Various Thresholds for Endogenous Endophthalmitis.

## Abbreviations

ANC	Absolute Neutrophil Count
AUC	Area Under the Curve
BCVA	Best-Corrected Visual Acuity
CI	Confidence Interval
CRP	C-Reactive Protein
DCA	Decision Curve Analysis
ESR	Erythrocyte Sedimentation Rate
IOP	Intraocular Pressure
IVI	Intravitreal Injection
logMAR	Logarithm of the Minimum Angle of Resolution
NPV	Negative Predictive Value
PCT	Procalcitonin
PPV	Positive Predictive Value
ROC	Receiver Operating Characteristic
SE	Standard Error
WBC	White Blood Cell Count
