# Supplementary material for: Serum Procalcitonin to Support Early Triage for Possible Systemic Infection in Patients with Endophthalmitis
Source: Diagnostics (Basel). 2026 Apr 29;16(9):1331. doi: 10.3390/diagnostics16091331 (PMC13164000; doi:10.3390/diagnostics16091331)
Supplement: Supplementary file 1 [file diagnostics-16-01331-s001.zip › diagnostics-4145499-supplementary.pdf]

Supplementary Table S1: Comparison of Included vs. Excluded Patients

Variable	Included (n=152)	Excluded (n=34)	p-value
Age (years)	67.00 (58-76)	65.50 (57-73)	0.456
Sex (Male %)	53.9% (82/152)	44.1% (15/34)	0.345
Diabetes Mellitus (%)	35.5% (54/152)	26.5% (9/34)	0.423
Initial BCVA (logMAR)	2.30 (1.30-2.30)	2.00 (0.30-2.30)	0.026
Hypopyon (%)	52.0% (79/152)	47.1% (16/34)	0.705
Panophthalmitis (%)	9.2% (14/152)	8.8% (3/34)	1.000
Endogenous Etiology	43.4% (66/152)	5.9% (2/34)	<0.001
Final BCVA (logMAR)	2.00 (0.35-3.00)	0.52 (0.15-2.30)	0.008

Supplementary table S2: Detailed information on the causative infections and pathogens

	Endogenous endophthalmitis (N=66)	Exogenous endophthalmitis (N=86)
Infection origins	Liver abscess (14)<br>Pneumoniae (9)<br>Urinary tract infection (6)<br>Soft tissue infection (6)<br>Colitis (3)<br>Infectious endocarditis (3)<br>Renal abscess (2)<br>Appendiceal abscess (1)<br>Cholecystitis (1)<br>Duodenal ulcer perforation (1)<br>Lung abscess (1)<br>Prostate abscess (1)<br>Spondylitis (1)<br>Unclear primary infection source (17)	Cataract surgery (31)<br>Corneal ulcer (18)<br>Penetrating trauma (17)<br>Bleb associated (6)<br>Vitrectomy (6)<br>Intravitreal injection (4)<br>Ahmed valve exposure (1)<br>Anterior chamber paracentesis (1)<br>ICL implantation (1)<br>Necrotizing scleritis (1)
Causative microorganism	*Klebsiella pneumonia* (17)<br>*Candida albicans* (8)<br>*Staphylococcus aureus* (5)<br>*Streptococcus agalactiae* (5)<br>*Candida tropicalis* (2)<br>*Enterococcus faecalis* (2)<br>*Candida parapsilosis* (1)<br>*Escherichia coli* (1)<br>*Streptococcus pneumonia* (1)<br>*Acinetobacter jejuni* &	*Staphylococcus epidermidis* (8)<br>*Enterococcus faecalis* (7)<br>*Pseudomonas aeruginosa* (3)<br>*Streptococcus pneumonia* (2)<br>*Streptococcus oralis* (2)<br>*Candida albicans* (1)<br>*Escherichia coli* (1)<br>*Staphylococcus haemolyticus* (1)<br>*Streptococcus agalactiae* (1)

	Staphylococcus epidermidis (2)<br>No growth (22)	Staphylococcus lugdunensis (1)<br>*Streptococcus salivarius* (1)<br>*Pluerostoma species* (1)<br>*Bacillus cereus* (1)<br>No growth (29)<br>No exam (27)

Supplementary Table S3. Diagnostic Performance of Procalcitonin at Various Thresholds for Endogenous Endophthalmitis

Threshold	Sensitivity (95% CI)	Specificity (95% CI)
0.05 ng/mL (normal range)	93.9% (83.5 - 97.9)	95.8% (86.0 - 98.8)
0.10 ng/mL (Angeletti, 2015)	91.8% (80.8 - 96.8)	95.8% (86.0 - 98.8)
0.11 ng/mL (ROC-derived)	91.8% (80.8 - 96.8)	97.9% (89.1 - 99.6)
0.25 ng/mL (Nieuwkoop, 2010)	81.6% (68.6 - 90.0)	97.9% (89.1 - 99.6)
0.50 ng/mL (Meisner, 2010)	73.5% (59.7 - 83.8)	100.0% (92.6 - 100.0)
